# The Effect of a 3-Minute Mindfulness Intervention, and the Mediating Role of Maximization, on Critical Incident Decision-Making

**DOI:** 10.3389/fpsyg.2021.674694

**Published:** 2021-05-28

**Authors:** Neil D. Shortland, Presley McGarry, Lisa Thompson, Catherine Stevens, Laurence J. Alison

**Affiliations:** ^1^Department of Criminology and Criminal Justice, University of Massachusetts Lowell, Lowell, MA, United States; ^2^Centre for Critical and Major Incident Psychology, University of Liverpool, Liverpool, United Kingdom

**Keywords:** decision-making, mindfulness, maximization, uncertainty, individual differences

## Abstract

**Objective:**

In this study, we extend the impact of mindfulness to the concept of least-worst decision-making. Least-worst decisions involve high-uncertainty and require the individual to choose between a number of potentially negative courses of action. Research is increasingly exploring least-worst decisions, and real-world events (such as the COVID-19 pandemic) show the need for individuals to overcome uncertainty and commit to a least-worst course of action. From sports to business, researchers are increasingly showing that “being mindful” has a range of positive performance-related benefits. We hypothesized that mindfulness would improve least-worst decision-making because it would increase self-reflection and value identification. However, we also hypothesized that trait maximization (the tendency to attempt to choose the “best” course of action) would negatively interact with mindfulness.

**Methods:**

Three hundred and ninety-eight participants were recruited using Amazon MTurk and exposed to a brief mindfulness intervention or a control intervention (listening to an audiobook). After this intervention, participants completed the Least-Worst Uncertain Choice Inventory for Emergency Responders (LUCIFER).

**Results:**

As hypothesized, mindfulness increased decision-making speed and approach-tendencies. Conversely, for high-maximizers, increased mindfulness caused a slowing of the decision-making process and led to more avoidant choices.

**Conclusions:**

This study shows the potential positive and negative consequences of mindfulness for least-worst decision-making, emphasizing the critical importance of individual differences when considering both the effect of mindfulness and interventions aimed at improving decision-making.

*“In the NBA, mindfulness is important because the game is chaotically fast and the pressures on players are extreme. In real life, it’s important because the practice can help us get a handle on ourselves and stop going into a tailspin or endless series of tangents.”*

Sirk (*Zen and the Art of Winning: Phil Jackson’s Team Leadership*, 2020).

## Introduction

Star athletes, from Michael Jordan, Simone Biles, Kobe Bryant, to Tiger Woods, are all associated with making crucial decisions at crucial times, and their legacies will be defined by their ability to successfully execute in these moments (Swann et al., 2017a,b; Schweickle et al., 2020). While the physical attributes of star athlete performance are often studied (e.g., Allen and Hopkins, 2015), psychologists have also extensively explored the psychological dimensions that underpin elite success (Mahoney and Avener, 1977). In reference to the athletes above, one now well-known aspect of their performance is the role of mindfulness in their preparation and playing of their game (Fink and Ruiz, 2020). Mindfulness–defined as focusing the attention on what is happening in the mind, body, or environment at that present moment in a non-judgmental or accepting way (Kabat-Zinn, 1991)–is a growing field of study within psychology and an increasing range of personal and interpersonal benefits of applying mindful practice are being discovered (Trent et al., 2016). Beyond athletics, mindfulness improves workplace learning (Hanson et al., 2020), eating disorders (Sala et al., 2020), veteran quality of life (Goldberg et al., 2020), and performance in university (Atkins et al., 2015). Crucial to this study is the emerging evidence that mindfulness enhances decision-making through improving executive functioning, attention, and emotional interference in cognitive tasks (Jha et al., 2007; Ortner et al., 2007). A range of gambling (Liu et al., 2014), experimental (Mirams et al., 2013), and ethical paradigms (Shapiro et al., 2012) have shown that mindfulness improves decision-making performance. In this study we seek to extend the study of mindfulness by exploring the degree to which mindfulness impacts a decision-makers ability to make high-stakes least-worst decisions that often manifest in domains defined by high-uncertainty such as emergency responses and military operations (van den Heuvel et al., 2012; Alison et al., 2015; Shortland et al., 2019, 2020a,b; Shortland and Alison, 2020).

### Least-Worst Decision-Making

The importance of least-worst decisions is evidenced by the scale of challenges faced in 2020. On one hand, governments across the world juggle the need to protect lives from the novel COVID-19 virus with the need to allow society (and the economy) to operate. At the same time, we have seen isolated incidents of police decision-making in civilian interactions lead to worldwide protests and calls to defund (and even disband) the police. From a psychological standpoint, what these two decision-making scenarios have in common is that the decision-maker must balance equally unappealing options concurrently with a need to choose between them (van den Heuvel et al., 2012; Alison et al., 2015; Shortland et al., 2019). In naturalistic decision-making, based on findings from research on decision-making in real-life critical incidents, researchers have called these types of decisions “least-worst” (see Power and Alison, 2017a,b). While least-worst decisions have received less attention than the more classic “rational” or “recognition” based decision-making strategies (Klein, 2011), the occurrence of such decisions in high-stakes situations, coupled with the tendency of individuals to avoid making least-worst decisions (Anderson, 2003; Alison et al., 2013; Power and Alison, 2017b; Shortland et al., 2019; Shortland and Alison, 2020) has spurred efforts to understand the psychological underpinnings of least-worst decision-making.

Least-worst decision-making can become “de-railed” in many ways. Firstly, individuals can become “inert” and suffer from redundant deliberation in which they fail to choose a course of action (Shortland and Alison, 2020). Secondly, individuals can choose an avoidant course of action to avoid potential blame for any negative outcome (van den Heuvel et al., 2014). These two complications that frequently arise with least-worst decision making are often augmented by the fear of making a deleterious or unpopular choice, or when individuals feel they lack sufficient information to conduct proper risk analyses. This is largely evident regarding the COVID-19 pandemic, where the hesitancy of political leaders to make least-worst decisions during extremely uncertain times lead to both increased spread of the virus, as well as increased fatalities (Hale et al., 2020). However, a range of naturalistic research has argued that *good* least-worst decision-making relies on the ability of the individual to triage competing goals and establish a clear hierarchy of values (i.e., knowing what the *single most* important outcome is, rather than identifying many important, but tangential outcomes). It is establishing this value hierarchy that allows individuals to completely, and without regret, commit to an underlying guiding goal/principle (Shortland et al., 2019; Shortland and Alison, 2020). Given this, there is significant warrant to hypothesize that mindfulness should facilitate the process of least-worst decision-making. Such as, it allows the individual to focus on the problem at hand, and to avoid becoming distracted by erroneous information and/or courses of action, and to be better aware of their own driving values (Kirk et al., 2016).

### Mindfulness

Research has shown that increased mindfulness has a range of positive health (such as lower psychological distress; Slemp et al., 2019) or minimized burnout (Iancu et al., 2018) and wider performance benefits (Vonderlin et al., 2020). For example, the core component of mindfulness–self-regulation of attention to focus on the present moment (Bishop et al., 2004)–allows individuals to better focus on a task by being less distractable by external stimuli (Good et al., 2016). Studies that have employed a mindfulness intervention have shown that mindfulness training improves attention, cognition, emotions, behavior, and physiology (Khoury et al., 2015; Spijkerman et al., 2016; Hendriks et al., 2017; Jayawardene et al., 2017). Mindfulness training has been shown to increase ethical decision-making (Ruedy and Schweitzer, 2010), improve moral reasoning (Shapiro et al., 2012), increase impulse control in relation to decisions to snack (Forman et al., 2016), and increase intentions to cooperate (Kirk et al., 2016). In addition to this, mindfulness is also said to increase curiosity and openness, and in doing so increase creativity, enhance problem-solving skills, and facilitate an individuals’ ability to cope with uncertainty (Jacobs and Blustein, 2008; Baas et al., 2014; Lebuda et al., 2016). All of these factors are critical to effective decision-making; especially least-worst decision-making (Shortland and Alison, 2020).

### Maximization

Making choices requires sacrifice in which the decision-maker knowingly rejects the benefits of one option in favor of the benefits of another. However, not all individuals are equally adept at making these sacrifices, especially when neither of the options are “ideal” (Dar-Nimrod et al., 2009; see also Schwartz, 2004). Maximization refers to the individual differences in peoples’ ability to sacrifice the “best” possible option for a satisfactory option that is “good enough”–according to their own threshold of acceptability (Schwartz, 2004). A range of studies have explored the effects of being a “maximizer,” showing that those who maximize are more likely to report low self-esteem overall (Schwartz et al., 2002), lower levels of happiness (Schwartz et al., 2002; Polman, 2010) and are less likely to be satisfied with their lives (Schwartz et al., 2002; Thompson and Dahling, 2012). Maximizers are also perfectionists (Schwartz et al., 2002; Bergman et al., 2007), greedier (Seuntjens et al., 2015), and more neurotic than satisficers (Purvis et al., 2011). In terms of their decision-making strategies, maximizers are less open (Purvis et al., 2011), more prone to procrastination (Osiurak et al., 2015), and are more likely to engage in counterfactual thinking (Schwartz et al., 2002). Furthermore, recent research has looked at the maximization tendencies of individuals who make least-worst decisions (which often require satisficing) and found that wider maximization tendencies also manifest in high-stakes decisions. Namely, maximizers found least-worst decisions harder and were slower to choose a course of action (Shortland et al., 2020a,b). While, to date, no research has explored the inter-section of maximization and mindfulness, there is ample warrant to assume that while mindfulness may improve least-worst decision-making, (a) mindfulness may make high maximizers *worse* at least-worst decision-making as the increased focus identifies more opportunities for an endless series of tangents and ruminations, or (b) mindfulness may override the effects of maximization by allowing the individual to focus on what is truly important, and thus not become stuck ruminating on what the “ideal” choice is.

### This Study

We hypothesize that mindfulness will improve least-worst decision-making by allowing individuals to be more objective and prevent redundant deliberation stemming from goal conflict between approach and avoidance goals (Alison et al., 2013, 2015; van den Heuvel et al., 2014; Power and Alison, 2017a,b; Shortland and Alison, 2020). Furthermore, we measure trait maximization, previously shown to influence least-worst decision-making (Shortland et al., 2020a,b) and hypothesize that individual differences in trait maximization will interact with a mindfulness intervention. Specifically, we hypothesize that those who are exposed to a mindfulness intervention will make decisions faster (H_1_), report a lower decision-making difficulty (H_2_), make more approach orientated decisions (H_3_) and that trait maximization will interact with the effect of a mindfulness intervention to improve least-worst decision-making (H_4_).

## Materials and Methods

### Participants

The sample for this study consists of three hundred and ninety-eight individuals were recruited from Amazon MTurk for this study. This sample went through two levels of screening. Firstly, the initial sample was 694 participants (i.e., all those who begun the study after being recruited from MTurk). Of this 694, only 404 completed all questions and scenarios. After a manual review of the data, a further six individuals were removed for evidencing behaviors indicative of poor quality (completing scenarios in an unrealistically short amount of time, answering all questions with the same response etc.). In addition to this, 48 outlier datapoints were identified and removed. These outliers presented with a lower bound of 1% and upper bound of 99% on any reaction time score. Forty-eight total scenario × participant data points were removed. This method allowed us to isolate single instances of potential distraction/poor data while maintaining the remaining datapoints for analysis.

This resulted in a final sample of 394 participants for analysis. Average age for the reduced sample (*N* = 398) was 40 (*M* = 40.71, *SD* = 12.73). Of these participants, 50.75% percent were male, and 51.26% of participants were randomly assigned to the experimental group. In accordance with best practices suggested by Difallah et al. (2018), we filtered our potential participant pool to include only United States residents with an MTurk approval rate of at least 95% (this approval rate represents the proportion of completed tasks that are approved by Requesters and is a recommended way to limit responses to only those Workers who have consistently produced high quality work; see also Clifford et al., 2015; Smith et al., 2015). Hauser and Schwarz (2016) demonstrated across three separate samples that MTurk workers are more attentive than their more traditional sample pool counterparts, and thus provide a suitable participant pool for this study. MTurk participants have also extensively been used to study mindfulness (e.g., Trent et al., 2016; Kappen et al., 2018; Klussman et al., 2020). This study involving human participants was reviewed and approved by an Institutional Review Board. Participants were required to provide their informed consent by selecting “I consent” before being allowed to continue with the study, individuals that chose to select “I do not consent” were re-directed to the end of the survey and thanked for their time and interest in the study.

### Procedure

All participants completed the measure of trait maximization (see Turner et al., 2012) with informed consent provided digitally prior to beginning the study. Participants were also reminded of their ability to end testing at any time and were asked to complete the battery in a single session. After completing the battery of psychometrics tests, participants were randomly assigned to the mindfulness or control intervention group. After exposure to either the mindfulness intervention (experimental group) or audiobook recording (control group), participants proceeded to complete a series of five decision-making scenarios [Least-Worst Uncertain Choice Inventory for Emergency Responders (LUCIFER); see below]. Post-decision-making, participants answered a range of scenario-specific attention checks before exposure to the debrief. The study procedure was approved by an institutional review board at a large northeastern university in the United States.

### Materials

#### Decision-Making

The current study uses the LUCIFER inventory of least-worst decisions first reported by Shortland et al. (2020b). LUFICER has been developed with support from ARI FSRU, the Combat Capabilities Development Command (CCDC) Soldier Center at Natick and the Center for Applied Brain and Cognitive Sciences at Tufts University and has been piloted with a range of applied groups (Soldiers, Police Officers, Fire Fighters, etc.). It has been used on over 1,000 practitioners across the United States and United Kingdom (Shortland et al., 2020a,b). LUCIFER adopts a 2-alternative forced choice (2AFC) approach in which individuals are presented with an audio-feed (recorded by members of the armed forces, or paid actors) that provide them with an assessment of the situation and a required action. All decisions were collected from first responders and military personnel who faced least-worst decisions in the field (see Shortland et al., 2019). Sample scenario scripts are included in Appendix A (see also Shortland et al., 2020b). LUCIFER operates through Qualtrics to allow accurate recording of response times. LUCIFER allows measurement of the following dependent variables, both on average across the scenarios, and per scenario):

(1) Situational Awareness Time (SAT): Time taken to listen to the decision context and declare they are “ready” to decide.

(2) Choice time (CT): Time taken to choose an option (A vs. B). This is measured as the time taken to record the last “click” on an option on the page (Qualtrics recorded both first and last page clicks for each step of the scenario meaning that a CT of 0 would reflect individuals that immediately “clicked” on their choice and moved forward to the next page of the survey without hesitation).

(3) Decision Time (DT): Time taken to choose and declare they are ready to “commit” (i.e., submit) their choice.

(4) Commitment Time (ComT): Time lag between selecting a course of action (CT) and committing to it (DT). In terms of calculation, ComT = DT–CT.

(5) Decision Difficulty (DD): Participants completed a five-item DD scale (see Hanselmann and Tanner, 2008) after completing each scenario.

(6) Approach/Avoidance (AA): Each LUCIFER scenario represents a choice between an approach decision (an active behavior that makes a positive impact) and an avoidance decision (no further behavior and to withdraw to prevent further harm. An overall AA score was calculated by summing the total number of approach choices made across all scenarios.

#### Mindfulness Intervention

A range of research has shown that even a short mindfulness intervention can result in quantifiable performance improvements. For example, in studies where a 10-min mindfulness intervention is applied, cooperation in group performance improves and the intervention group reported increases in openness, open-mindedness, perceived importance (Cleirigh and Greaney, 2015) and compassion (Fernando et al., 2017). A meta-analysis of 65 randomized-control trials that used a brief mindfulness intervention found a small but significant effect of brief mindfulness training on reducing negative affect (Schumer et al., 2018). This study employed the 3-min breathing space (3MB), which involves (a) focusing awareness of present internal experiences; (b) focusing awareness on the breath; and (c) expanding awareness to the body as a whole. The 3MB intervention was chosen because it has been used as part of a range of previous mindfulness interventions (either in isolation or as part of a wider intervention package, e.g., King et al., 2013). Participants in the control group were asked to listen to a brief segment of an audiobook (*Harry Potter* read by Stephen Fry) which was matched in length to 3MB. Additionally, this task was designed so that participants could still exit the survey at any time but could not advance within the survey before the full 3MB task or audiobook segment had been played.

#### Maximization

Maximization was measured using Turner et al. (2012) 34-item Maximization Inventory. Each item is scored using a six-point scale ranging from “Strongly Disagree” (1) to “Strongly Agree” (6). The maximization scale involves three components of maximization: satisficing (10 items), DD (12 items), and alternative search (12 items). Satisficing measures the degree to which someone chooses outcomes that reach the threshold of “acceptability,” rather than ones much closer to optimal. This subscale includes items such as, “At some point you need to make a decision about things.” DD measures the frustration–or difficulty–that one experiences when making a choice. Example items in this subscale include, “I am usually worried about making a wrong decision.” Finally, alternative search measures an individual’s tendency to seek all available options before committing to a choice. Items in this subscale include, “I take the time to consider all alternatives before making a decision.”

## Results

### Overall Performance

On average, participants took 25 s to understand the situation and declare themselves “ready” to decide (SAT; *M* = 24.55, *SD* = 15.38; see Table 1). Typically, participants took just under 6 s to decide (DT; *M* = 5.80, *SD* = 6.16), which includes the total time to make and then commit to a choice. Meaning the initial choice took participants, on average, 5 s (CT; *M* = 4.54, *SD* = 5.84), while committing to this decision usually took participants 1 s (ComT; *M* = 1.26, *SD* = 1.74). The most common scenario difficulty score given was “medium” (*M* = 50.90, *SD* = 17.27), and there was a tendency to make “Approach” choices (*M* = 6.24, *SD* = 1.94).

**TABLE 1:** Descriptive statistics for participants (*N* = 398).

Descriptive Statistics (*N* = 398)				

	Mean	St. Deviation	Min.	Max.
***Outcome Variable (N* = *3932)***				
Situational Awareness Time (SAT)	24.55	15.38	0.66	132.06
Choice Time (CT)	4.54	5.84	0.81	106.43
Decision Time (DT)	5.80	6.16	1.10	107.45
Commitment Time (ComT)	1.26	1.74	0.10	68.03
Decision Difficulty (DD)	50.90	17.27	0.40	100.50
Approach Score (AA)	6.244	1.94	0.0	10.00
***Individual Level Variables (N* = *398)***				
Maximization–Satisficing	48.33	7.38	17.00	60.00
Maximization–Decision Difficulty	40.80	12.51	12.00	72.00
Maximization–Alternative Search	49.94	11.04	12.00	72.00
Age	40.71	12.73	18.00	77.00

	*N*	%		

**Gender**				
Male	202	50.75		
Female	196	49.25		
**Experimental**				
Yes	204	51.26		
No	194	48.74		

Maximization was examined as three subscales: satisficing (max_sat), DD (max_diff), and alternative search (max_alt). Participants’ satisficing scores ranged from 17.0 to 60.0 (*M* = 48.33, *SD* = 7.38). DD subscale scores ranged from 12.0 to 72.0 (*M* = 40.80, *SD* = 12.51), and alternative search scores ranged from 12.0 to 72.0 (*M* = 49.94, *SD* = 11.04).

Preliminary analyses also included a series of Pearson’s correlations which indicated that the Maximization subscale scores significantly correlated to some of the model variables. Specifically, the satisficing subscale was positively associated with SAT (*r* = 0.07, *n* = 3932, *p* < 0.001), DT (*r* = 0.04, *n* = 3932, *p* = 0.025), and ComT (*r* = 0.04, *n* = 3932, *p* = 0.009) and negatively associated with DD (*r* = −0.05, *n* = 3932, *p* < 0.001). The DD subscale was positively associated with DD (*r* = 0.15, *n* = 3932, *p* < 0.001) and AA (*r* = 0.21, *n* = 3932, *p* < 0.001). The alternative search subscale was positively associated with DD (*r* = 0.08, *n* = 3932, *p* < 0.001) and AA (*r* = 0.07, *n* = 3932, *p* < 0.001), but negatively associated with being in the experimental group (*r* = −0.06, *n* = 3932, *p* < 0.001).

### Group Differences

A series of *t*-tests were run to test individual group differences between the control group and the experimental group for each of the model’s variables. For every reaction time type, the experimental group exhibited faster reaction times than the control group (see Table 2). The experimental group (*M* = 51.69, *SD* = 17.08) also reported slightly higher levels of DD compared to the control group (*M* = 50.16, *SD* = 17.40). The control group (*M* = 6.24, *SD* = 1.93) reported a slightly lower tendency to approach than the experimental group (*M* = 6.25, *SD* = 1.95). Of the maximization scales, the experimental group reported higher scores than the control group for the satisficing and DD subscales but reported a lower score for the alternative search subscale than the control group.

**TABLE 2:** Group differences for performance between control and experimental groups.

	All Participants	Control Group (*n* = 2020)	Experimental Group (*n* = 1912)	Mean Difference
**SAT** (seconds)	24.55 (15.38)	27.28 (32.13)	24.05 (14.61)	3.23 (*p* < 0.001)
**CT** (seconds)	4.54 (5.84)	4.95 (10.92)	4.38 (4.70)	0.57 (*p* < 0.001)
**DT** (seconds)	5.80 (6.16)	6.22 (11.14)	5.62 (4.99)	0.60 (*p* < 0.001)
**ComT** (seconds)	1.26 (1.74)	1.27 (2.16)	1.25 (1.11)	0.02 (*p* < 0.001)
**DD**	50.90 (17.27)	50.16 (17.40)	51.69 (17.08)	1.53 (*p* < 0.001)
**AA**	6.24 (1.94)	6.24 (1.93)	6.25 (1.95)	0.01 (*p* < 0.001)
**Age** (years)	40.71 (12.73)	39.26 (12.39)	42.26 (12.88)	3.00 (*p* < 0.001)

### Multi-Level Modeling

Multi-level modeling (MLM) was used to test the effect of the Maximization subscales and the role of the mindfulness intervention on decision-making, while controlling for age and gender. MLM for each of the dependent variables organized the total 3980 data points both by the random effects of the five two-step scenarios (i.e., 10 decision points) and by the 398 participants, resulting in a final *N* = 3980 data points used in analyses. Using this structure, a two-level MLM was used to estimate the main effect of Maximization on SAT, DT, CT, ComT, DD, and AA (see Table 3), as well as the interactive effect of Maximization and experimental group. The outcome of these MLMs is displayed in Table 3.

**TABLE 3:** Multi-level model outcomes for all variables (*N* = 3932).

Models	β	SE	Odds Ratio [Exp. (β)]	*p*
***1. Situational Awareness (N* = *3932)***				
Constant	16.262	5.5000	1.155 × 10^7^	0.003
Max_Sat	0.209	0.104	1.232	0.044*
Max_Diff	0.083	0.060	1.086	0.169
Max_Alt	–0.116	0.077	0.891	0.132
Age	0.041	0.040	1.041	0.313
Gender (Male = 1)	–0.597	0.914	0.505	0.514
Experimental (Yes = 1)	0.006	6.832	1.006	0.999
Max_Sat × Experimental	–0.041	0.141	0.960	0.772
Max_Diff × Experimental	–0.109	0.082	0.897	0.185
Max_Alt × Experimental	0.105	0.104	1.110	0.314
***2. Choice Time (N* = *3932)***				
Constant	3.953	1.487	52.009	0.008
Max_Sat	–0.009	0.029	0.991	0.748
Max_Diff	0.038	0.017	1.038	0.027*
Max_Alt	–0.020	0.022	0.980	0.360
Age	0.021	0.011	1.021	0.064
Gender (Male = 1)	–0.295	0.259	0.745	0.256
Experimental (Yes = 1)	–1.804	1.942	0.165	0.353
Max_Sat × Experimental	0.065	0.040	1.067	0.105
Max_Diff × Experimental	–0.045	0.023	0.956	0.053
Max_Alt × Experimental	0.002	0.029	1.002	0.950
***3. Decision Time (N* = *3932)***				
Constant	4.608	1.683	100.258	0.006
Max_Sat	–0.006	0.033	0.994	0.867
Max_Diff	0.042	0.019	1.042	0.030*
Max_Alt	–0.018	0.025	0.982	0.463
Age	0.026	0.013	1.026	0.046*
Gender (Male = 1)	–0.338	0.294	0.713	0.251
Experimental (Yes = 1)	–1.847	2.201	0.158	0.401
Max_Sat × Experimental	0.071	0.045	1.073	0.119
Max_Diff × Experimental	–0.050	0.026	0.951	0.057
Max_Alt × Experimental	0.001	0.033	1.001	0.986
***4. Commitment Time (N* = *3932)***				
Constant	0.656	0.400	1.927	0.101
Max_Sat	0.004	0.008	1.004	0.607
Max_Diff	0.004	0.005	1.004	0.395
Max_Alt	0.002	0.006	1.002	0.732
Age	0.004	0.003	1.004	0.153
Gender (Male = 1)	–0.044	0.071	0.956	0.531
Experimental (Yes = 1)	–0.022	0.532	0.978	0.966
Max_Sat × Experimental	0.005	0.011	1.005	0.642
Max_Diff × Experimental	–0.005	0.006	0.995	0.433
Max_Alt × Experimental	–0.001	0.008	0.999	0.877
***5. Decision Difficulty (N* = *3932)***				
Constant	47.763	7.015	5.536 × 10^20^	0.000
Max_Sat	–0.086	0.141	0.917	0.540
Max_Diff	0.278	0.082	1.321	0.001***
Max_Alt	–0.046	0.104	0.955	0.657
Age	0.026	0.055	1.027	0.629
Gender (Male = 1)	–6.648	1.241	0.001	0.000***
Experimental (Yes = 1)	9.811	9.268	1.823 × 10^4^	0.290
Max_Sat × Experimental	–0.233	0.191	0.792	0.222
Max_Diff × Experimental	–0.203	0.112	0.816	0.069
Max_Alt × Experimental	0.225	0.141	1.252	0.111
***6. Approach Score (N* = *3932)***				
Constant	3.250	0.765	25.782	0.000
Max_Sat	0.030	0.007	0.007	0.000***
Max_Diff	0.0477	0.004	1.049	0.000***
Max_Alt	–0.014	0.005	0.986	0.006**
Age	0.003	0.003	1.003	0.224
Gender (Male = 1)	0.551	0.061	1.735	0.000***
Experimental (Yes = 1)	1.491	0.450	4.443	0.001***
Max_Sat × Experimental	–0.031	0.009	0.970	0.001***
Max_Diff × Experimental	–0.017	0.005	0.983	0.002**
Max_Alt × Experimental	0.013	0.007	1.013	0.057

*p < 0.05; **p < 0.01; ***p < 0.001.

Across each of the models outlined in Table 3, maximization does appear to be statistically significantly linked to CT, decision time, DD, and tendency to approach, while inclusion in the group that received the intervention appears to have a significant impact on tendency to approach. Furthermore, there also appears to be an interactive effect between at least one maximization subscale and inclusion in the experimental group that significantly impacts CT, decision time, and tendency to approach.

Specifically, there is a positive association between max_sat and SAT (β = 0.209, *p* = 0.044). Additionally, there is a positive association between max_diff and CT, with CT increasing by an average of 0.04 s (*p* = 0.02) for every one-point increase on the max_diff subscale, controlling for all other variables and interaction effects. There was no significant interaction effect for maximization scores and experimental group.

There is a positive association between max_diff and decision time, with decision time increasing by an average of 0.04 s (*p* = 0.03) for every one-point increase on the max_diff subscale, controlling for all other variables and interaction effects. Additionally, decision time was found to increase by 0.026 for every 1-year increase in age (*p* = 0.046). Again, there was no significant interaction effect for maximization scores and experimental group.

There is a positive association between max_diff and DD scores, with DD increasing by an average of 0.27 points (*p* < 0.001) for every one-point increase on the max_diff subscale, controlling for all other variables and interaction effects. Additionally, males are less likely to report higher DD scores (OR = 1.41 × 10^–3^, *p* < 0.001), compared to female participants.

**FIGURE 1:**
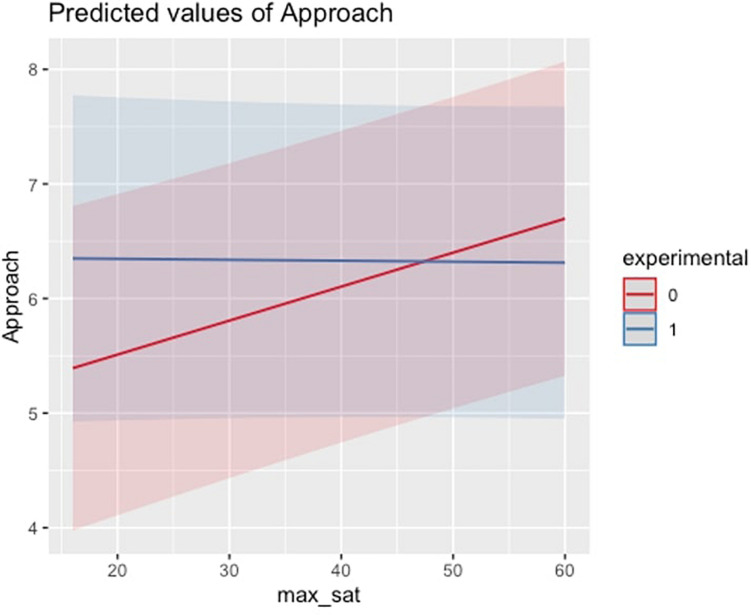
Interaction effect for Approach Score and Maximization (satisficing subscale).

Lastly, there are several variables that have a statistically significant effect on tendency to approach. There is a positive association between max_sat and approach score, with approach score increasing by an average of 0.03 points (*p* < 0.001) for every one-point increase on the max_sat subscale, controlling for all other variables and interaction effects. What is interesting here is that while approach score remained relatively static regardless of max_sat score for the experimental group, approach score appears to increase as max_sat score increases for the control group (see Figure 1).

**FIGURE 2:**
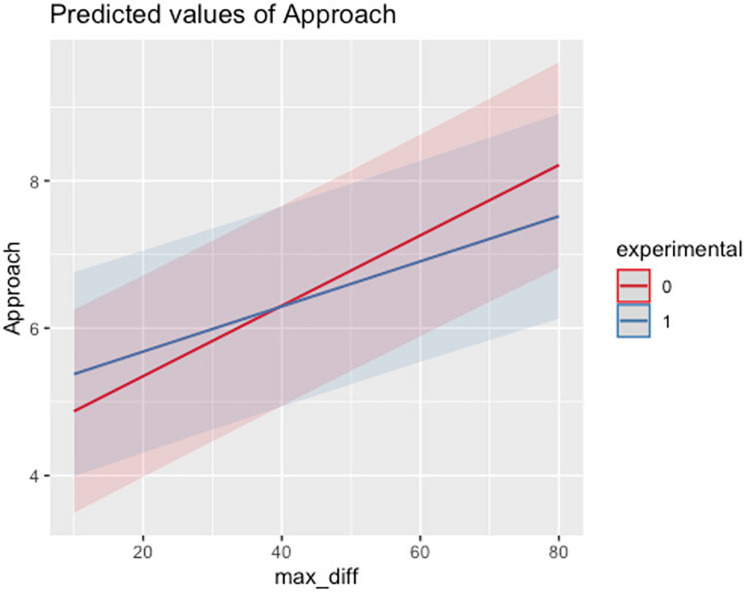
Interaction effect for Approach Score and Maximization (decision difficulty subscale).

There is also a positive association between max_diff and approach score, with approach score increasing by an average of 0.05 points (*p* < 0.001) for every one-point increase on the max_diff subscale, controlling for all other variables and interaction effects. In addition to the significant main effect of max_diff on approach score, the difference in approach scores between the experimental and control groups narrows as max_diff scores increase and then after reaching a score of about 40 the between group difference in approach score begins to widen again (see Figure 2).

There is also a negative association between max_alt and approach score, with approach score decreasing by an average of 0.01 points (*p* = 0.006) for every one-point increase on the max_alt subscale, controlling for all other variables and interaction effects. Males are more likely to report higher approach scores (OR = 1.74, *p* < 0.001), compared to female participants. Participants in the experimental group are more likely to report higher approach scores (OR = 4.44, *p* = 0.001), compared to participants in the control group.

Overall, results indicate that the effect of maximization was dependent on experimental group for approach score. For the group that received the mindfulness intervention, approach scores appeared to remain static for varying levels of max_sat score. However, for the control group, approach scores increase as max_sat scores increase. Additionally, for both the control and experimental group, increases in max_diff was associated with increases in approach score; however, an increase in max_diff corresponded to a larger increase in approach score for the control group when compared to the experimental group. These results indicate that those exposed to a mindfulness intervention had a higher approach score than individuals in the control group for individuals low in maximization score. However, as maximization score increased, approach scores were higher for individuals in the control group when compared to the experimental group.

## Discussion

Critical incidents, as with sports, are chaotically fast, the pressure on decision-makers is extreme, and the outcomes of these critical events depends on the ability of the decision-maker to overcome uncertainty and risk to make critical decisions in the hopes of achieving the best outcomes. As such, in this study we hypothesized that exposure to a brief mindfulness intervention (3MB) would improve the ability of individuals to make least-worst decisions. Furthermore, we hypothesized that maximization (the trait tendency to seek the “best” choice rather than satisficing for one that is “good enough”; Schwartz et al., 2002) would interact with the mindfulness intervention. Overall, we found support for several of our hypotheses. While those exposed to a mindfulness intervention did not find the decisions easier, or make decisions faster, they were more approach oriented in their decision-making. Furthermore, as hypothesized, trait maximization interacted with the effect of the mindfulness intervention. Specifically, those who scored higher on the satisficing and the DD subscale of maximization were also more likely to choose an avoidant course of action after being exposed to the mindfulness intervention. These findings are critically important because they show that mindfulness has the potential to serve as a double-edged sword depending on the personality traits of those who are exposed to it. We discuss the theoretical and practical implications of these findings below.

While a range of research has focused on the process of least-worst decision-making (Power and Alison, 2017a,b) and even the personality traits that may be associated with good least-worst decision-making (Shortland et al., 2020a,b), this research was the first to explore the effect of an intervention which aimed to improve least-worst decision-making. The effect of mindfulness is increasingly being explored: from improving expert sports performance to the workplace (Fink and Ruiz, 2020), and here we showed that a brief mindfulness intervention has the ability to potentially improve least-worst decision-making in certain individuals. What is especially interesting is that while the individuals exposed to 3MB were more likely to choose approach orientated decisions, previous research has found that avoidance-based decisions are caused by accountogenic fears which stem from worries about blame (Waring et al., 2013; van den Heuvel et al., 2014). Elsewhere, mindfulness training has been shown to increase ethical decision-making (Ruedy and Schweitzer, 2010), improve moral reasoning and ethical decision-making (Shapiro et al., 2012). Perhaps in the current study, mindfulness led to a better ability to focus on the needs of others over the accountogenic fears of the self. Another viable explanation is that because 3MB was administered before the decisions, and the effect lasted when performance was analyzed across five two-step scenarios, mindfulness improved attention, cognition, emotions, behavior, and physiology (Khoury et al., 2015; Spijkerman et al., 2016; Hendriks et al., 2017; Jayawardene et al., 2017), decreased the immediate impulse to withdraw (Forman et al., 2016), and/or increased participant intentions to cooperate (Kirk et al., 2016). While this study is not able to define the specific route through which mindfulness led to approach tendencies, it provides evidence that a short mindfulness intervention created a quantifiable shift in decision-making tendencies across a range of diverse least-worst decisions.

Despite the positive benefit of the mindfulness intervention, the effect was not universal. Mindfulness made high maximizers more likely to choose avoidant outcomes. Individual differences in the tendency to maximize has been studied extensively in the wider psychological literature on choice and decision-making (e.g., Parker et al., 2007; Sparks et al., 2012). Research has specifically found that maximizers are slower to commit to choices and find least-worst decisions more difficult (Shortland et al., 2020a,b). The findings here are critically important because they show (1) that mindfulness interventions have the potential to decrease performance for certain individuals and (2) that future research on the effect of mindfulness on decision-making (or wider behaviors) needs to measure relevant personality traits that could interact with the psychological processes that accompany a state of mindfulness. Here, mindfulness is thought to improve focus on the present moment (Bishop et al., 2004), and in doing so, allow one to better focus on a task (Good et al., 2016). However, it is viable that this is a recipe for worsening the decision-making of maximizers. Specifically, Parker et al. (2007) found that self-reported maximizers stated greater spontaneity in their decision-making making “spur-of-the-moment choices.” This spontaneity was correlated with reporting decision avoidance; meaning that those who postponed making decisions ended up making what felt like “spur of the moment” choices (Parker et al., 2007).

In this study, it is viable to propose that mindfulness forced focus onto the task at hand, in which participants then found themselves attempting to maximize a decision that has no “ideal” outcome resulting in a slowing of the decision-making process. Maximizers showed increased avoidance in relation to their low-maximization and non-mindful peers. While the specific route through which maximization led to an increase in avoidance after exposure to a mindfulness intervention cannot be delineated with this study design, it makes an important point: that the effect of mindfulness is likely an interaction with individual personality, and in some instances, mindfulness can be a double-edged sword–equally improving and worsening performance based on the trait tendencies of the subject. This highlights the significance of tailoring intervention strategies to the individuals participating in them, as our results support the notion that the practice of mindfulness could benefit the decision-making capabilities for some, while being detrimental for other individuals with certain personality traits, such as high maximization. Thus, this research indicates the importance of creating and implementing evidence-based interventions that take into consideration individual differences in personality. Further research is required which expands on these assertions to further unpack the interaction of mindfulness, maximization and decision-making.

## Conclusion

At the time of writing, the world is contending with the novel COVID-19 pandemic, and the successful management and mitigation of the virus is based on effective least-worst decision-making at a every level. From political leaders deciding if, how, and when to “lockdown” their country, to medical professionals deciding how to triage patients and how (and to whom) to distribute the first doses of the vaccine. In many ways COVID-19 has shown the critical importance of the ability to overcome uncertainty, doubt, and multiple competing potentially negative courses of action (Shortland et al., 2020a,b). In the response to COVID-19, just as in many other critical incidents (such as responding to humanitarian crises or terrorist attacks; Alison et al., 2013), what it critical is avoiding decision inertia (“doing nothing”; Anderson, 2003) overcoming fear of accountability or blame and committing to courses of action that will best aid the situation (van den Heuvel et al., 2014). In this study we sought to demonstrate the importance of a brief mindfulness activity that could, realistically, improve the ability of the decision-maker to focus internally, establish an internal hierarchy, and commit to a least-worst choice. While extensive further research is required in this area, the results of this study do support that least-worst decision-making can be improved, and that activities that encourage self-reflection may provide a route to do this. However, of critical importance, the effect of mindfulness is not homogenous, whilst mindfulness improved decision-making in some, in others is created an environment in which least-worst decision-making was slowed and became more avoidant. These findings highlight several important theoretical and practical points, which could not be more important given the global circumstances. Specifically, that a relatively short mindfulness intervention improved people’s ability to make incredibly hard decisions. This is an incredibly exciting proposition that demonstrates just one example of a technologically simple, low-cost, and highly available tool that can be implemented to improve decision-making in the situations where it matters most. To date, we try and solve decision-making with training and technology, even going so far as to explore artificial intelligence and brain-computer interfaces. Here, decision-making was improved with a simple mindfulness activity, and while we cannot ignore the important moderating role of personality, this research advocates that in the face of immense pressure to add increasingly impressive technology to our decision-making processes, self-reflection and mindful breathing may continue to play an equally, if not more, important role.

## Data Availability Statement

The raw data supporting the conclusions of this article will be made available by the authors, without undue reservation.

## Ethics Statement

The studies involving human participants were reviewed and approved by University of Massachusetts Lowell, Institutional Review Board. The patients/participants provided their written informed consent to participate in this study.

## Author Contributions

NS conceptualized the empirical study, developed the research methodology, oversaw recruitment, and wrote the manuscript. PM, LT, and CS oversaw the running of the empirical study, data cleaning, hypothesis testing, and results write-up. LA provided oversight, reviewed the manuscript, and assisted in the design of the experimental paradigm. All authors contributed to the article and approved the submitted version.

## Conflict of Interest

The authors declare that the research was conducted in the absence of any commercial or financial relationships that could be construed as a potential conflict of interest.

## Appendix A

### Tunnel Scenario

Hi Commander, there has been an explosion in Merseyside tunnel we have deployed the Police and Fire Services there now to help with evacuations and casualties. The problem is that we’re hearing rumors that there is a secondary bomb in the tunnel which could go off at any time. We don’t have a lot more information on the source, except the security services say it is a “credible threat.” Do you want to call the officers out of the tunnel?

### Second Step:

“Sir, one of the officers is refusing to come out of the tunnel. He says he is with an 8 year old girl who is trapped. Her mums dead but she’s only pinned in by some metal. He needs some pedal cutters to get her out and he won’t come out until he’s got those cutters. Some of the other officers have volunteered to go back with the cutters. Shall I let them go?

### Mountain Fire Scenario

Sarge, Private First Class Billings here. I am here with some Special Forces guys. They say they have intelligence about the base of operations for the local insurgents who have been targeting our base with missiles over the past month. The intel comes from an insurgents’ brother. He says they have a base of operations at about 11000 feet in some nearby mountains. We’ve got UAV footage of the area. It’s pretty grainy, but there is definitely some movement up there. They are likely to move by morning, so the window is time limited. They are saying this is our chance. Do you want me to mobilize air support?”

### Second Step:

[Call from Squad Commander]. I’ve just heard from PFC Billings. What the hell are you doing. We are looking at the exact same intelligence as you! Undo your decision right now.

### Rescue Mission

Captain, we have just heard from our guys in the field, they were being dropped by helicopter at a landing sight up in the mountains and they came under heavy fire. They aborted the landing but one of the officers fell out of the helicopter in all the confusion. The status of soldier is unknown. The helicopter has landed in a safe spot on the valley floor but is requesting that they immediately turn around and go back to the landing zone to retrieve their Soldier.”

### Second Step:

“We have received a satellite feed from the ground that shows the Landing Zone where the Soldier was last seen. The footage is unclear but appears to show him in and amongst a lot of enemy soldiers. We cannot confirm if he is free and fighting, captured, or has since been killed. The other guys still want to go after him. What do you want to do?”
